# The development and validation of a CT-based radiomics signature for the preoperative discrimination of stage I-II and stage III-IV colorectal cancer

**DOI:** 10.18632/oncotarget.8919

**Published:** 2016-04-22

**Authors:** Cuishan Liang, Yanqi Huang, Lan He, Xin Chen, Zelan Ma, Di Dong, Jie Tian, Changhong Liang, Zaiyi Liu

**Affiliations:** ^1^ Department of Radiology, Guangdong General Hospital, Guangdong Academy of Medical Sciences, Guangzhou, 510080, China; ^2^ Graduate College, Southern Medical University, Guangzhou, 510515, China; ^3^ School of Medicine, South China University of Technology, Guangzhou, Guangdong, 510006, China; ^4^ Department of Radiology, The Affiliated Guangzhou First People’ Hospital, Guangzhou Medical University, Guangzhou, 510180, China; ^5^ Key Laboratory of Molecular Imaging, Chinese Academy of Sciences, Beijing, 100190, China

**Keywords:** colorectal cancer, computed tomography, radiomics signature, predictor, stage

## Abstract

**Objectives:**

To investigative the predictive ability of radiomics signature for preoperative staging (I-II*vs.*III-IV) of primary colorectal cancer (CRC).

**Methods:**

This study consisted of 494 consecutive patients (training dataset: n=286; validation cohort, n=208) with stage I–IV CRC. A radiomics signature was generated using LASSO logistic regression model. Association between radiomics signature and CRC staging was explored. The classification performance of the radiomics signature was explored with respect to the receiver operating characteristics(ROC) curve.

**Results:**

The 16-feature-based radiomics signature was an independent predictor for staging of CRC, which could successfully categorize CRC into stage I-II and III-IV (*p* <0.0001) in training and validation dataset. The median of radiomics signature of stage III-IV was higher than stage I-II in the training and validation dataset. As for the classification performance of the radiomics signature in CRC staging, the AUC was 0.792(95%CI:0.741-0.853) with sensitivity of 0.629 and specificity of 0.874. The signature in the validation dataset obtained an AUC of 0.708(95%CI:0.698-0.718) with sensitivity of 0.611 and specificity of 0.680.

**Conclusions:**

A radiomics signature was developed and validated to be a significant predictor for discrimination of stage I-II from III-IV CRC, which may serve as a complementary tool for the preoperative tumor staging in CRC.

## INTRODUCTION

Colorectal cancer (CRC), as the second most commonly cancer in females and the third in males, has estimated 1.4 million cases and led to 693,900 deaths in 2012 [1]. For early-stage (stage I and II) colorectal cancer, resection surgery is regard as the most common treatment option; while chemotherapy is generally the main treatment option for patients with advanced-stage (stage III and IV) colorectal cancer [2]. The 5-year survival rate of colorectal cancers detected at localized stage is 90.3%, while it drops to 70.4% when the cancer involves lymph nodes or adjacent organs [2]. Therefore, accurate preoperative staging to distinguish early-stage CRCs from advanced-stage CRCs is essential for treatment planning strategy [3, 4].

In clinical practice, computed tomography (CT) has been commonly used to assist the treatment planning strategy for CRC patients. However, the accuracy of pre-treatment staging by CT varies, ranging from 48% to 98% in different reports [5–12]. Recent studies have found that based on widely available CT images, texture analysis could provide additional information reflecting the underlying biologic heterogeneity [13, 14]. As for the tumor staging, CT-based texture analysis has recently been demonstrated to be predictive in esophageal cancer and non-small cell lung cancer (NSCLC) [15, 16]. Although those texture features, as individual predictors, have been proved to be significantly associated with tumor staging, the combined analysis of a panel of multiple predictors as a signature has nowadays been regarded as a more powerful method to assist clinical management [17]. Radiomics, which enables a high-throughput extraction of quantitative features from medical images, has facilitated the construction of powerful signatures [18, 19].

To date, to the best of our knowledge, scarcely any study has conducted with radiomics signature to predict the staging of primary colorectal cancer. Therefore, the aim of this study is to investigative the predictive ability of radiomics signature based on CT image for the preoperative staging (I-II vs. III-IV) of primary colorectal cancer.

## RESULTS

### Clinicopathologic characteristics of patients

494 patients who met the inclusion criteria were divided into two groups including training dataset with 286 patients between November 2005 to April 2009 and the validation dataset with 208 patients from May 2009 to December 2010. The clinicopathologic characteristics of these patients in the training and validation dataset are listed in Table 1. There was no difference between the training dataset and the validation dataset in the clinicopathologic characteristics (*p* = 0.419-0.546).

**Table 1:** Characteristic of CRC patients in the training dataset and the validation dataset

Characteristic	Training dataset	Validation dataset	P value
**Gender(No[%])**			
Male	189 (66.1)	132 (63.5)	0.546
Female	97 (33.9)	76 (36.5)	
**Age(yr, m [r])**	63 (19-90)	64 (27-88)	0.524
**Stage(No[%])**			
I-II	127 (44.4)	100 (48.1)	0.419
III-IV	159 (55.6)	108 (51.9)	

Note: CRC, colorectal cancer; No, number; yr, year; m, median; r, range.

P value < 0.05 indicates a significant difference in patients’ characteristic between the training dataset and validation dataset.

### Radiomics signature building

16 features were selected using the lasso logistic regression model (Supplementary Material S1). The radiomics signature score (Rad-score) was calculated for each patient based on these features as follows: *Rad – score* = 5.62223376 + contrast_0_0 × 0.0861 − homogeneity_0_0 × 0.960 + skewness_1.0 × 4.30 − contrast_135_2.5 × 0.120-homogeneity_45_2.5 × 0.430 − his_50_SD_2.5 × 0.0000196 + his_10_mean_2.5 × 0.000318 + his_10_SD_2.5 × 0.0000361 − correlation_45_1.5 × 1.50 + correlation_135_1.5 × 4.80 + his_25_mean_1.5 × 0.00370 + skewness_2.0 × 0.913-homogeneity_0_1.0 × 10.3 + his_50_mean_1.0 × 0.00123-his_25_SD_1.0 × 0.000000310 + skewness_1.5 × 1.06

### Predictive performance

#### Predictive performance of the radiomics signature

The median value of radiomics signature in the training dataset and validation dataset was listed in Table 2. There's significant difference between the median of radiomics signature of stage III-IV patients and that of the stage I-II patients, in both the training dataset (p < 0.0001) and the validation dataset (p < 0.0001, with the former much higher.) The radiomics signature presented good performance for the discrimination of stage I-II patients and stage III-IV patients, which yielded an AUC of 0.792 in the training dataset and 0.708 in the validation dataset (Table 3).

**Table 2:** The Rad-score for the training dataset and validation dataset

Rad-score	Stage I-II	Stage III-IV	p - value
	Median (IQR)	Median (IQR)	
**Training dataset**	−0.0741(−0.256,0.0684)	0.188(0.0145,0.425)	<0.0001
**Validation dataset**	−0.00382(−0.158,0.162)	0.188(−0.00269,0.327)	<0.0001
**Gender**			
Male	−0.040(−0.375,0.287)	0.521(0.056,1.086)	<0.001
Female	0.043(−0.462,0.413)	0.659(0.088,1.089)	<0.001
**Age**			
Age<=65yr	0.010(−0.452,0.287)	0.514(0.074,0.983)	<0.001
Age>65yr	0.021(−0.340,0.360)	0.618(0.051,1.226)	<0.001
**Histological grade**			
Poorly differentiated	0.009(−0.388,0.313)	0.574(0.051,1.085)	<0.001
Well-moderately differentiated	0.107(−0.307,0.380)	0.587(0.323,1.090)	0.004

Note: IQR, interquartile range; p – value < 0.05 indicates a significant difference in the median Rad-score between stage I-II and stage III-IV CRC patients.

**Table 3:** Predictive performance of the radiomics signature, maximum diameter and clinical model

Variables & model	Training dataset						Validation dataset				
	Cutoff	AUC (95%CI)	SEN	SPE	Accuracy	P Value	AUC (95%CI)	SEN	SPE	Accuracy	P Value
Diameter	4.500	0.590 (0.582, 0.598)	0.566	0.559	0.563		0.554 (0.435,0.457)	0.519	0.450	0.486	
Signature	0.392	0.792 (0.741,0.853)	0.629	0.874	0.738	<0.001^ψ^	0.708 (0.698,0.718)	0.611	0.680	0.644	0.006^ψ^
Clinical model	0.205	0.632 (0.624, 0.640)	0.528	0.701	0.605	<0.001^ξ^	0.592 (0.581, 0.603)	0.472	0.670	0.567	0.037^ξ^
Combined model	0.263	0.811 (0.805,0.817)	0.723	0.803	0.759	<0.001^η^	0.719 (0.709,0.729)	0.750	0.580	0.668	0.006^η^

Note: 95%CI: 95% confidence interval. AUC: area under curve. SEN: sensitivity; SPE: specificity. ψ, ξ, η were calculated by Delong test for the comparison of AUC between radiomics signature and maximum diameter, between radiomics signature and clinical model, and between combined model and clinical model, respectively. p – value < 0.05 indicates a significant difference of AUC between radiomics signature and maximum diameter, between radiomics signature and clinical model, and between combined model and clinical model, respectively.

**Figure 1:**
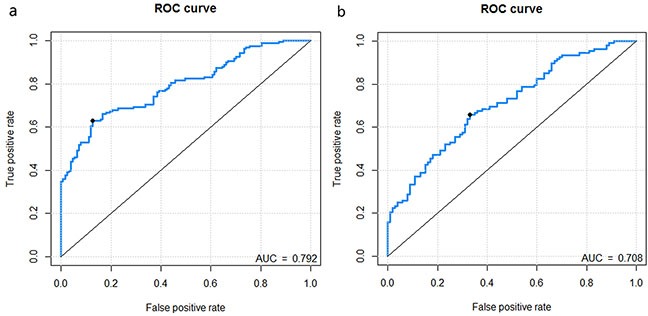
Receiver operating characteristic (ROC) curves of the radiomics signature in the training dataset and validation dataset **Figure 1a-1b.** represents the ROC curves of radiomics signature for training dataset, validation dataset, respectively.

**Figure 2:**
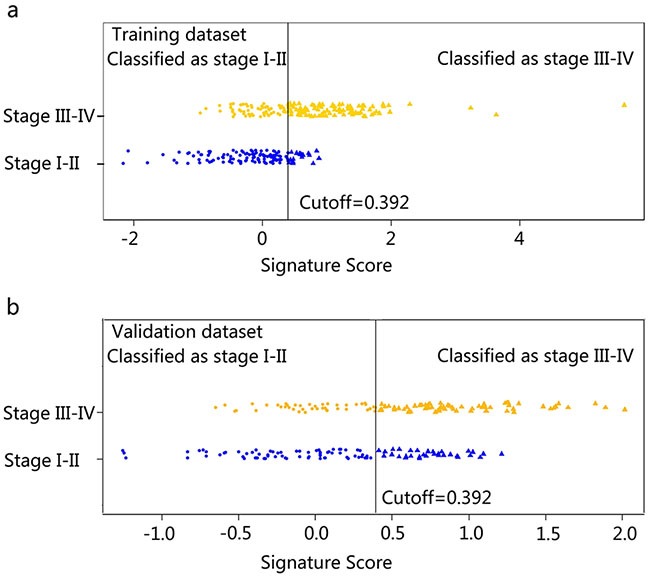
Signature scores for each patient regarding the classification of tumor stage (I-II vs. III-IV) in the training dataset and validation dataset **Figure 2a-2b.** represents the signature scores distribution in the training (2a) and validation (2b) dataset. The blue marks indicate stage I-II CRC patients, while the gold marks indicate stage III-IV CRC patients. The solid line presents the best cutoff of radiomics signature for the discrimination of stage I-II and stage III-IV CRC patients, below which patients are discriminated to be stage I-II CRC patients and above which patients are discriminated to be CRC stage III-IV patients. The cutoff value is 0.392.

The predictive performance for the classification of stage I-II vs. III-IV CRC in the training and validation dataset presented by ROC were described in Figure 1a and Figure 1b. The Rad-scores for each patient in the training and validation dataset regarding the classification of stage I-II vs. III-IV CRC were depicted in Figure 2a and Figure 2b.

#### Predictive performance of the maximum diameter and clinical model

The AUCs of the maximum diameter and the clinical model were 0.554 and 0.592 in the validation dataset, respectively. The predictive performance (sensitivity, specificity and accuracy) of the maximum diameter and clinical model was shown in Table 3.

**Figure 3:**
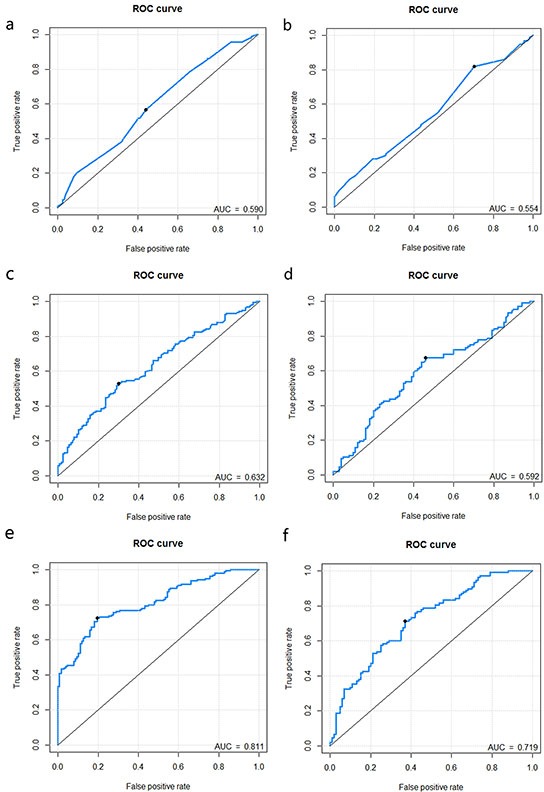
Receiver operating characteristic (ROC) curves of the maximum diameter, clinical model and combined model **Figure 3a-3f.** represents the ROC curves of maximum diameter (training: Figure 3a.; validation: Figure 3b.), clinical model (training: Figure 3c.; validation: Figure 3d.) and combined model (training: Figure 3e.; validation: Figure 3f.).

The predictive performance for the classification of stage I-II vs. III-IV CRC of the maximum diameter, the clinical model in the training and validation dataset presented as ROC were described in Figure 3.

#### Comparison of predictive performance between radiomics signature and maximum diameter

Delong test showed that there was significant difference in the staging performance between the radiomics signature and maximum diameter either in the training dataset (p< 0.001) or in the validation dataset (p = 0.006), with the radiomics signature showing better performance (AUC in the validation dataset: 0.708 vs. 0.554).

#### Added value of the radiomics signature to the clinical model

Compared with the clinical model, the radiomics signature showed significantly better performance, either in the training dataset (AUC: 0.792 vs. 0.632; p< 0.001) or in the validation dataset (AUC: 0.708 vs.0.592; p = 0.037).

The combined model showed better staging performance compared with the clinical model (AUC in validation dataset: 0.719 vs. 0.592; p = 0.006) (Table 3).

#### Stratified analysis for the radiomics signature in CRC staging

**Figure 4:**
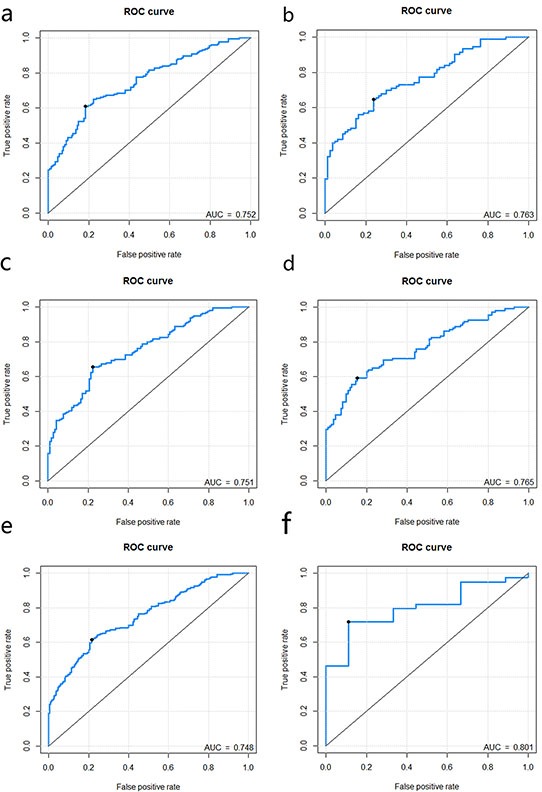
**Figure 4a-4f.** represents the ROC curves of radiomics signature for each subgroup when stratified by gender (male: Figure 4a.; female: Figure 4b.), age (<=65: Figure 4c.; >65: Figure 4d.), histological grade (poorly differentiated: 4e; well-moderately differentiated: 4f).

**Figure 5:**
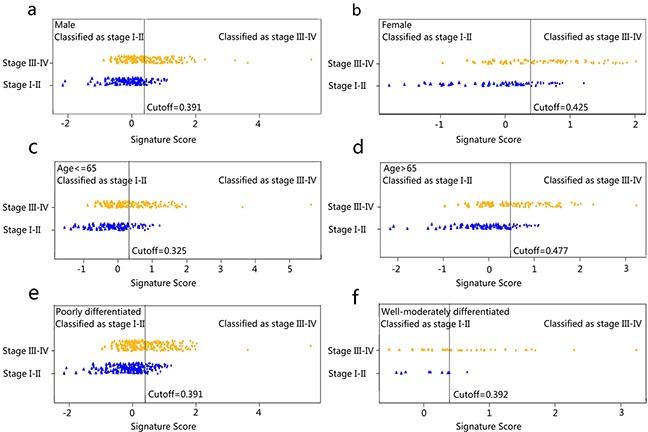
Signature scores for each patient regarding the classification of tumor stage (I-II vs. III-IV) in subgroups The blue marks indicate stage I-II CRC patients, while the gold marks indicate stage III-IV CRC patients. The solid line presents the best cutoff of radiomics signature for the discrimination of stage I-II and stage III-IV CRC patients, below which patients are discriminated to be stage I-II CRC patients and above which patients are discriminated to be CRC stage III-IV patients. The cutoff values for the discrimination in subgroups were as follow: male, 0.391; female, 0.425; age <= 65, 0.325; age > 65, 0.477; poorly differentiated, 0.391; well-moderately differentiated, 0.392, respectively).

Furthermore, the stratified analysis showed that the constructed radiomics signature was still an independent predictor for the discrimination of stage I-II and stage III-IV CRC patient even after adjusting for gender, age, and histological grade (p = 0.004; Table 2). The radiomics signature presented good performance for the discrimination of stage I-II patients and stage III-IV patients in the subgroups. (Table 4). The predictive performance for the discrimination of stage I-II and III-IV CRC within subgroups presented as ROC were described in Figure 4. The signature scores for each patient within subgroups regarding the classification of stage I-II vs. III-IV CRC were depicted in Figure 5a–5f.

**Table 4:** Predictive performance of the radiomics signature stratified by gender, age, and histological

Variables	Cutoff	AUC	95%CI	SEN	SPN	Accuracy
**Gender**						
Male	0.391	0.752	(0.746, 0.758)	0.609	0.816	0.704
Femal	0.425	0.763	(0.753, 0.773)	0.645	0.763	0.699
**Age**						
<=65	0.325	0.751	(0.744, 0.758)	0.654	0.778	0.707
>65	0.477	0.765	(0.756, 0.772)	0.593	0.846	0.720
**Histological grade**						
Poorly differentiated	0.391	0.748	(0.743, 0.751)	0.614	0.784	0.697
Well-moderately differentiated	0.392	0.801	(0.763, 0.839)	0.718	0.889	0.750

Note: 95%CI: 95% confidence interval. AUC: area under curve. SEN: sensitivity; SPE: specificity.

## DISCUSSION

In this study, a novel 16-feature based radiomics signature was developed and validated to be an independent predictor for the discrimination of stage I-II and stage III-IV CRC. Furthermore, in the subgroup stratified by gender, age and histological grade, the radiomics signature was also manifested as a useful satisfied predictor in discriminating stage I-II from stage III-IV CRC. Our results demonstrated that the radiomics signature could successfully categorize CRC patients into stage I-II and stage III-IV, with large differences in Rad-scores.

In clinical practice, imaging modalities such as magnetic resonance imaging (MRI), positron emission tomography (PET)/CT and computed tomography (CT), which can provide both anatomic and morphologic information of tumors, are commonly used for the staging in CRC patient. [4] Although MRI is recommended by the American college of radiology for the T staging of CRC patients [20], the difficulty to differentiate tumor infiltration from fibrosis limited their ability to distinguish stage T2 tumors from early stage T3 tumors [4]. Besides, a major drawback limiting the application of MRI in preoperative assessment is that this imaging modality is expensive as well as time consuming, let alone the potential motion artifact. PET/CT is valuable for the assessment of distant metastases. However, PET/CT is also expensive and time consuming. In addition, physiological fluorodeoxyglucose gastrointestinal uptake may give rise to misinterpretation [4]. As for the preoperative CT, though the accuracy is considerably high in T staging [21], it is poorer in N staging [7, 12].

In patients with colorectal cancer, analysis of liver texture on portal phase CT images has provided preliminary evidence to be a superior predictor of survival than CT perfusion imaging and a poorer 5-year overall survival rate found to be associated with fine-texture features [13, 22]. Texture features were also demonstrated to be associated with stage of other cancers. For example, in the study of Dong et al, all metabolic parameters of PET/CT were correlated significantly with T stage of American Joint Commission on Cancer (AJCC) in patients with esophageal squamous cell carcinoma [23]. Fine texture features on unenhanced computed tomography to identify tumors above stage II were demonstrated to correlate with tumor stage [15]. Though texture feature -based analysis is an individual predictor, it has been proved to be a valuable, potential biomarker for diagnosis, prediction and prognosis based the evidences presented above.

In this study, rather than focusing on individual texture feature, the novel analysis of a panel of features as a signature was presented. Recently, radiomics signature has been demonstrated to be a significant predictor for the survival in oropharyngeal squamous cell carcinoma (p = 0.004), a prognostic biomarker for distant metastasis for lung adenocarcinoma patients (AUC = 0.61), and a classifier associated with the staging in lung, head and neck cancer [24–26]. In the latter study, the reported highest AUC for the staging of lung cancer was 0.61, which is much lower than our study (AUC =0.792); while the AUC for that in the head tumor was 0.77, which was comparable to our study (AUC = 0.792 for training dataset; AUC = 0.708 for the validation dataset). Similarly, radiomics signature was demonstrated to have prognostic capacity for overall survival in patients with lung and head-and-neck cancer, with an AUC of 0.65 for lung cancer and 0.69 for head-and-neck cancer [27]. Indeed, the combined analysis of a panel of multiple predictors as a signature has nowadays been regarded as the most promising method to assist clinical practice [17]. Compare to the analysis of individual texture features for the prediction conducted in CRC [13, 28], the predictive radiomics signature presented in this study may provide more comprehensive predictive information for clinic practice. In our study, the radiomics signature was demonstrated to be an independent predictor for discriminate stage I-II from stage III-IV in both the training and validation datasets.

Although tumor size has been reported to show discrimination ability for the staging of several tumors [29, 30], to date there has been scarcely any study demonstrated the predictive performance of tumor maximum diameter for discriminating stage I-II and stage III-IV CRC. As a measurement that could be obtained from CT image, it is of interest whether the maximum diameter could facilitate the CRC staging. As shown in this study, the maximum diameter of the tumor showed worse discrimination performance compared with the constructed radiomics signature. The unsatisfactory predictive performance of the maximum diameter for staging is not surprising since unlike the solid tumor, CRC lesions are relatively flexible that grows with no preferred direction, which may result in various tumor shape and even inaccuracy in the maximum diameter measuring. Therefore, with the concern that the addition of the maximum diameter into the combined clinical model may introduce bias, it was not integrated into the combined clinical.

Furthermore, the radiomics signature surpassed the discrimination performance of the clinical model and showed complementary performance to the clinical variables when added into the combined model.

Unlike other prior investigations that discriminated stage I-III from stage IV or stage I from stage II-IV of CRC, our current study focused on distinguishing stage I-II from stage III- IV, as the therapeutic strategy and outcome are significant different between these two groups [2]. Though there hasn't been any study to investigate the predictive ability of radiomics signature in discriminating these two groups, in the previous studies, other significant predictors such as total serum RNA and the serum proteomic have already been proved to be associated with tumor stage in the CRC [31, 32]. As an alternative and noninvasive tool, the radiomics signature presented in this study can provide additional predictive information that may be easy-to-use in clinical practice in the future.

The limitation of this study is that only 2-dimentional (2D) analysis on the largest axial slice was applied. Considering the selected largest single cross-sectional slice may not adequately represent heterogeneous characteristics of CRC, it is of interest that whether a 3-dimentional (3D) analysis may present better performance. Although there are several studies presented with the conclusion that the 3D analysis appeared more representative of tumor heterogeneity [28]. However, the 3D whole tumor analysis is computationally more complex and time-consuming. Besides, the study of Lubner et al showed that there was no significant difference between texture results of 2D and 3D analysis, with the conclusion that a single slice-2D texture analysis being adequate [33]. Therefore, we decided to performed 2D analysis as an initial attempt. Further studies exploring the potential usefulness of 3D radiomics analysis for the staging of colorectal cancer is of interest. In conclusion, in our study, a radiomics signature was developed and validated to be a significant predictor for the discrimination of stage I-II from stage III-IV CRC. As a noninvasive examination method and a potential imaging biomarker, radiomics signature provides a clinically valuable approach to identify individual characteristics and guide the preoperative staging, which is of great significance for the individual therapeutic strategy.

## MATERIALS AND METHODS

### Patients

On account of this was a retrospective study, informed patient consent was a waiver. However, it was still approved by our institutional review board. 859 consecutive patients diagnosed with CRC at our institute were recruited retrospectively between November 2005 and December 2010. The inclusion criteria for our study were: (1) biopsy-proven untreated CRC. (2) visible CRC on preoperative CT. (3) surgical resection with histopathological confirmation. Patients without preoperative CT (n=157); no surgery (n=85) on account of advanced disease or who chose to get treated elsewhere and only accepted a CT examination at our institute; preoperative chemotherapy (n=123) were excluded. The final study of 494 patients divided into two groups including training dataset with 286 patients [189 males (66.1 %), 97 females (33.9%)] with a median age of 63 years (range 19 to 90 years) who underwent pelvic contrast enhanced portal venous CT between November 2005 to April 2009 and the validation dataset with 208 patients [132 males (63.5 %), 76 females (36.5 %)] with a median age of 64 years (range 27 to 88 years) who underwent contrast-enhanced CT between May 2009 to December 2010. All tumors were adenocarcinomas (see Table 1). Histologic examination was confirmed by colorectal endoscopic biopsy, which was made by a professional gastrointestinal histopathologist. The age at diagnosis and gender were retrieved from the institution archive.

### Assessment of tumor stage

Tumor stage was established postoperatively by surgical oncologists and defined according to the 7th edition of the AJCC TNM staging system [3].

### CT imaging protocol

All patients underwent abdominal and pelvic contrast enhanced arterial and portal venous CT using either of the two multi-detector row CT (MDCT) unit (GE Lightspeed Ultra 8, GE Healthcare, Hino, Japan or 64-slice LightSpeed VCT, GE Medical systems, Milwaukee, Wis). The utilized parameters are given below: 120 kV; 130 mAs; 0.6- or 0.4s rotation time; detector collimation: 8×0.625mm or 64×0.625mm; field of view, 350×350mm; matrix, 512×512. Following routine noncontrast-enhanced CT, arterial- and portal-venous phase contrast-enhanced CT were performed after 20 s and 60 s of delay after intravenous injection of 90-100 ml of iodinated contrast material (Ultravist 370, Bayer Schering Pharma, Berlin, Germany) at a rate of 3.0 to 3.5 ml/s with a pump injector (Ulrich CT Plus 150, Ulrich Medical, Ulm, Germany). All the arterial- and portal-venous phase CT was reconstructed with 2.5 mm of reconstruction thickness.

### CT assessment of tumor maximum diameter

Tumor maximum diameter was assessed using curved planar reformation. A curved line was drawn along the center of the affected bowel on a stack of axial, sagittal, coronal section at a GE workstation (GE Medical systems, Milwaukee, Wis). Several curved planes were displayed and the maximum diameter of the tumor was measured.

### Texture analysis

The portal-venous phase CT was retrieved from the PACS (picture archiving and communication system) (Carestream, Canada), because of well differentiation between tumor tissue and adjacent normal bowel wall, followed by texture features extraction using in-house texture analysis algorithms applied in Matlab 2010a (Mathworks, Natick, USA).On the largest cross-sectional area, a region of interest (ROI) was delineated around the tumor outline by the exclusion of the air areas with attenuation values below −50 HU by a radiologist with 12 years of experience in abdominal CT interpretation, who was blinded to the clinical outcome.

### Image filtering

A process was applied to selectively extract features of diverse sizes and intensity variations. A Laplacian of Gaussian spatial band-pass filter (∇^2^*G*) was used, by turning the filter parameter between 1.0 and 1.5. The filter values of 0 indicated no filtration, 1.0 indicated degrees of fine texture, 1.5 and 2.0 indicated medium textures, while 2.5 indicated coarse texture. The corresponding Matlab code was attached as Supplementary Material S2. The Laplacian of Gaussian filter (∇^2^*G*) distribution is given by

$${}^{2}\text{G}(\text{X,Y})=\frac{-1}{\pi \pi \sigma^{4}}(1-\frac{{\text{x}}^{2}+{\text{y}}^{2}}{{2\sigma }^{2}}{)\text{e}}^{-(\frac{{\text{x}}^{2}+{\text{y}}^{2}}{{2\sigma }^{2}})}$$

x, y denote the spatial coordinates of the pixel and σ is the value of filter parameter.

### Feature generation

150 texture features were extracted from a single 2-dimensional CT slice, including the features from the category of gray-level co-occurrence matrix (GLCM) and histogram. (Supplementary Material S3)

### Statistical analyses

#### Feature selection and radiomics signature building

To select the most valuable prognostic features, the least absolute shrinkage and selection operator method (LASSO) logistic regression model [34], capable for the high dimensional data regression, was applied in the training dataset.

Through the linear combination of selected features multiplying by their respective coefficients, the radiomics score (Rad-score) was calculated for each patient.

### Predictive performance

#### Predictive performance of the radiomics signature

Mann–Whitney U test was used to estimate the relationship between radiomics signature and CRC staging (I-II and III-IV). To evaluate the classification ability of the radiomics signature, the receiver operating characteristics (ROC) curves were then generated in the training dataset. The optimal cutoff threshold values were determined at the point on the ROC curve at which the positive likelihood ratio (true positive fraction/false positive fraction) was maximal, followed by the derivation of the sensitivity and specificity [35]. The selected ROC cutoffs of the radiomics signature in the training dataset were then applied to the validation dataset to derive the sensitivity and specificity. Area under the curve (AUC) and diagnostic accuracy to distinguish the staging was derived in both the training dataset and validation dataset. The distribution of the signature scores of each patient regarding the classification of tumor stage was depicted.

#### Predictive performance of the maximum diameter and clinical model

A clinical prediction model including the CEA level, gender, and age was built based on the Logistic regression model. Besides, a combined model integrating the radiomics signature, CEA level, gender, and age was then built based on the Logistics regression model.

The classification ability of the clinical model and combined model were assessed, with ROC curves generated and classification measures (AUC, sensitivity, specificity, accuracy) derived.

#### Comparison of predictive performance between radiomics signature and maximum diameter

The comparison of the predictive performance (AUC) between the maximum diameter and radiomics signature was assessed by Delong test [36].

#### Added value of the radiomics signature to the clinical model

The comparison of the predictive performance (AUC) between the clinical model and the radiomics signature was assessed by Delong test. The added value of the radiomics signature to the clinical model was assessed through the comparison of the AUC of the combined model and that of the radiomics signature.

#### Stratified analysis for the radiomics signature

With the concern that there may be confounding within the derived results [37], we present a stratified analysis by the gender, age and histological grade.

Lasso logistic regression was done using the “glmnet” package using R software, version 3.0.1 (http://www.Rproject.org). Other statistical analysis was performed with SPSS for Windows, version 20.0 (IBM, Armonk, NY, USA). A two-sided p value was always computed, and a difference was considered statistically significant at p < 0.05.

## SUPPLEMENTARY MATERIALS


