# Supplementary material for: The development and validation of a CT-based radiomics signature for the preoperative discrimination of stage I-II and stage III-IV colorectal cancer
Source: Oncotarget. 2016 Apr 22;7(21):31401–12. doi: 10.18632/oncotarget.8919 (PMC5058766; doi:10.18632/oncotarget.8919)
Supplement: Supplementary file 1 [file oncotarget-07-31401-s001.pdf]

SUPPLEMENTARY MATERIAL

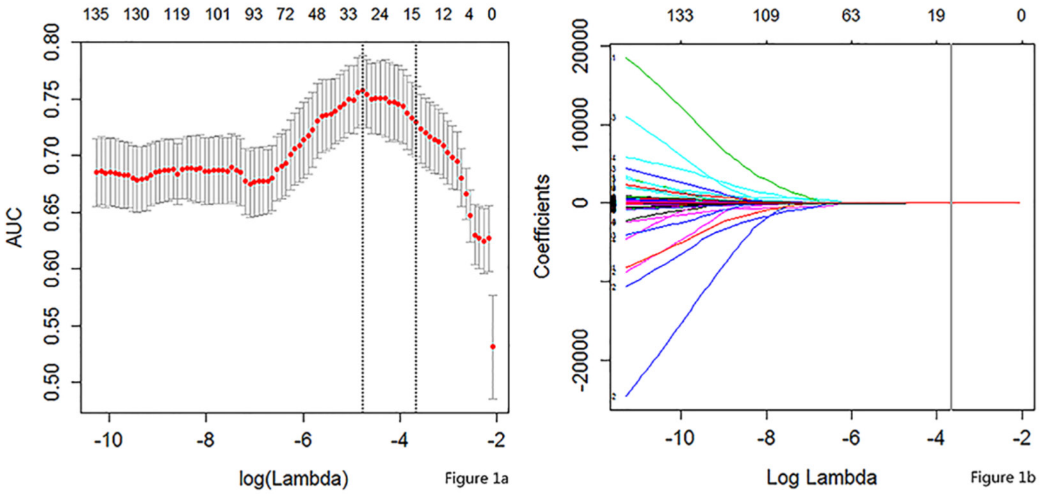

Supplementary Material S1: Texture feature selection using the LASSO logistic model. 16 features were selected using the lasso logistic regression model. Using the LASSO logistic model, the AUC was drawn versus log (λ). The optimal values (the vertical lines) of the lasso tuning parameter (λ) can be chosen using either the 1-SE criteria or the minimum criteria by generalized cross-validation. In this study, λ was selected based on the tenfold cross-validation used the 1-SE criteria that yielded a subset selection of 16 texture features, since the AUC (AUC>0.70) was similar to that when choosing features based on the minimum criteria (AUC>0.70).

```
%lapFilter.m
function im_filt = lapFilter(im, win, sigma)
% im: CT image; win=5;sigma=0,1.0,1.5,2.0,2.5;
if mod(win,2) == 0
    win = win+1;
end
hwin = (win-1) / 2;
% Laplacian Gaussian filter
filter = zeros(win);
for i = -hwin : hwin
    for j = -hwin : hwin
        exponent = -(i.^2+j.^2) / (2*sigma.^2);
        filter(i+hwin+1, j+hwin+1) = -1 / (pi*sigma.^4) * (1+exponent)*exp(exponent);
    end
end
im_filt = im;
[nrow ncol] = size(im);
for i = 1+hwin:ncol-hwin
    for j = 1+hwin:nrow-hwin
         tmp = im(i-hwin:i+hwin, j-hwin:j+hwin) .* filter;
        im_filt(i, j) = sum(tmp(:));
    end
end
end
```

Supplementary Material S2: Matlab code for image filtering.

Gray-level histogram	
Mean measures the average value of the histogram; *SD* measures the stability of the gray level histogram	$mean = \frac{1}{N}\sum_{i=1}^{N} X(i)$ $SD = \frac{1}{N}\sum_{i=1}^{N}(X(i) - \bar{X})^2$
Percentile mean and *SD* measures are calculated from the top 50%, 25%, and 10% of the histogram curve	$mean_\beta = \frac{1}{N-M}\sum_{i=M}^{N} X(i)$ $SD_\beta = \frac{1}{N-M}\sum_{i=M}^{N}(X(i) - \bar{X})^2$
Kurtosis describes the sharpness of the histogram	$kurtosis = \frac{\frac{1}{N}\sum_{i=1}^{N}(X(i) - \bar{X})^4}{\left(\sqrt{\frac{1}{N}\sum_{i=1}^{N}(X(i) - \bar{X})^2}\right)^4}$
Skewness describes the degree of asymmetry around the mean value in the gray level histogram	$skewness = \frac{\frac{1}{N}\sum_{i=1}^{N}(X(i) - \bar{X})^3}{\left(\sqrt{\frac{1}{N}\sum_{i=1}^{N}(X(i) - \bar{X})^2}\right)^3}$
Gray-Level Co-Occurrence Matrix (GLCM)	
Contrast measures local intensity variation, reflects the uniformity of image grayscale distribution and the degree of thickness in texture:	$contrast = \sum_{i=1}^{N_g}\sum_{j=1}^{N_g} \lvert i-j \rvert^2 P(i,j)$
Correlation measures the gray level linear dependence between the pixels at the specified positions relative to each other:	$correlation = \frac{\sum_{i=1}^{N_g}\sum_{j=1}^{N_g} ijP(i,j) - \mu_i(i)\mu_j(j)}{\sigma_x(i)\sigma_y(j)}$
Entropy describes the irregularity of an image.	$entropy = -\sum_{i=1}^{N_g}\sum_{j=1}^{N_g} P(i,j)\log[P(i,j)]$
Uniformity is the sum of squares of entries in the GLCM, measures the image homogeneity:	$uniformity = \sum_{i=1}^{N_g}\sum_{j=1}^{N_g}[P(i,j)]^2$
Homogeneity weights as the inverse of the Contrast weight:	$homogeneity = \sum_{i=1}^{N_g}\sum_{j=1}^{N_g}\frac{P(i,j)}{1+\lvert i-j \rvert^2}$

Supplementary Material S3: The textural features from the category of gray-level co-occurrence matrix (GLCM) and histogram.
